# A Latin American perspective on microbiome research

**DOI:** 10.1038/s41467-025-66756-y

**Published:** 2025-11-26

**Authors:** Alejandro Reyes, Claudio Durán, Silvia Rodríguez-Otálora, Deborah Delgado Pugley, Gregorio Iraola, Maria Gloria Domínguez-Bello, Trevor D. Lawley, Pablo Tsukayama

**Affiliations:** 1 Universidad de los Andes, Bogotá, Colombia; 2 Wellcome Sanger Institute, Hinxton, UK; 3 Pontificia Universidad Católica del Perú, Lima, Peru; 4 Kinzbio, Montevideo, Uruguay; 5 Rutgers University, New Brunswick, NJ USA; 6 Universidad Peruana Cayetano Heredia, Lima, Peru

**Keywords:** Microbiome, Metagenomics

## Abstract

The human gut microbiome plays a crucial role in human health, adapting and responding to changes in diet, environment, and lifestyle. However, current microbiome research is heavily biased toward high-income countries, leaving regions such as Latin America and the Caribbean (LAC) severely underrepresented. This imbalance limits our global understanding of microbial diversity and hinders the development of region-specific health interventions. In this Perspective, we discuss how LAC offers an exceptional opportunity for microbiome studies due to its unique ethnic diversity, rapid urbanization, distinct dietary traditions, and dual burden of infectious and chronic diseases. We highlight key findings from regional microbiome research, emphasizing the high diversity of ancestral microbial communities and the rapid shifts occurring in response to urbanization and globalization. To address existing disparities, we also introduce the Latinbiota Consortium, a collaborative network formed to strengthen local scientific capacity, ensure ethical and equitable research practices, promote data sovereignty, and foster inclusive participation by LAC researchers within global microbiome science. Through strategic investment and international collaboration, Latinbiota aims to preserve microbial diversity and ensure equitable participation in global microbiome science.

## Abstract (web summary)

In this Perspective, the authors provide a view on the state of the microbiome research in Latin America and the Caribbean, and present the Latinbiota Consortium, a collaborative network to foster inclusive participation by regional researchers within global microbiome science.

## Introduction

The human microbiome—the collection of bacteria, archaea, fungi, and viruses that inhabit our bodies—functions as a dynamic, rapid-response system, constantly adapting to environmental changes^1,2^. It serves as a barrier from our surroundings, interacting directly with diet, pathogens, pollutants, and numerous ecological exposures. Its immense genetic repertoire enables us to respond rapidly to environmental shifts beyond what our genome alone could achieve. Far from isolated, the microbiome exists along continuous gradients shaped by our environment and social interactions.

The gut microbiome, in particular, is highly responsive to dietary patterns, lifestyle, water quality, and exposures to animals and environments^3,4^. Comprehensive mapping of these variables is critical for defining meaningful “healthy” baselines and understanding dysbiosis in context. The development of tailored and effective microbiome-based therapies cannot be achieved without robust data from diverse populations and regions, particularly those that have been historically underrepresented.

Latin America and the Caribbean (LAC) remain significantly underrepresented in microbiome research, contributing only about 3% of global microbiome datasets while representing roughly 8.4% of the worldwide population^5,6^. This disparity limits our understanding of global microbial diversity and the development of region-specific health interventions. Despite significant limitations, studies based in LAC have explored microbiome diversity in Indigenous communities, tracked rapid microbiome shifts in urbanizing areas, and investigated health challenges that remain understudied in high-income settings^7–11^. These contributions show that the region is key to answering fundamental scientific questions.

The potential for future microbiome discovery in LAC remains vast. The region’s ancestral, ecological, and cultural diversity, combined with rapid transitions in urbanization, diet, and disease burden, offers unique conditions for studying microbial function, adaptation, and resilience. LAC is not just a gap in the knowledge, but a testbed for global questions that cannot be answered elsewhere. Recognizing this opportunity, the *Latinbiota Consortium* was launched as a regional initiative aimed at coordinating and catalyzing inclusive microbiome research across the LAC region, thereby bridging gaps in representation, equity, and scientific discovery.

## Unique opportunities Of Latin America and the Caribbean

With exceptional ethnogeographic and dietary diversity, Latin America and the Caribbean (LAC) provides a uniquely valuable setting for advancing microbiome science. Across diverse ecosystems—from Amazonian rainforests and Andean highlands to densely populated urban centers—humans maintain distinct diets and lifestyles that enable direct comparisons between traditional and industrialized populations^4,10,12,13^. These contrasts, often observed within a single country, reveal how geography, subsistence strategies, and food cultures shape the gut microbiome.

Local diets rich in maize, beans, tropical fruits, and fermented foods likely support microbial configurations rarely found in Western populations^4,7,10,14^.

LAC is also a key region for One Health research, where human, animal, and environmental interactions are particularly dynamic. In rural, coastal, and forested areas, close and continuous contact with both domestic animals and wildlife facilitates microbial exchange across species^8,15^. This ecological interface, combined with high biodiversity and rapid land-use change, offers opportunities to study microbiome transmission, pathogen spillover, and ecosystem resilience. Moreover, social structures such as kinship and co-residence networks have been shown to influence microbial sharing within communities, offering insights not readily accessible in more industrialized settings^16,17^.

The region’s human populations are highly diverse, shaped by Indigenous, European, African, and Asian ancestries. This genomic admixture provides a valuable opportunity to study host–microbiome interactions across varied genetic backgrounds. Traditional knowledge—including the use of medicinal plants, food fermentation, and community-based health practices—may also reveal microbiome management strategies refined over generations.

Microbiome research in LAC also holds promise for addressing the region’s dual burden of disease. Undernutrition, parasitic infections, and diarrheal diseases persist alongside rising rates of obesity and metabolic disorders^9,13,18^. These overlapping health challenges are increasingly linked to the gut microbiome but remain poorly studied in the regional context. Locally generated data could inform more effective, culturally relevant interventions than those based on high-income country models.

Despite these opportunities, microbiome research in the region faces significant challenges, including chronic underfunding and underrepresentation in global datasets. Urbanization and globalization are rapidly changing diets and lifestyles, threatening ancestral microbial communities and driving key species towards extinction. This situation highlights the urgent need to invest in microbiome science in LAC to preserve microbial diversity and to develop therapies tailored to specific contexts. It is also vital to ensure that global microbiome research encompasses the full range of human and environmental variation. Strengthening regional networks and fostering equitable international partnerships will be crucial to achieving this goal.

## Insights from LAC microbiome studies

Meta-analyses of human gut microbiome datasets consistently highlight the severe underrepresentation of Latin America and the Caribbean. For example, among 11,850 shotgun metagenomes analyzed by Almeida et al., fewer than 100 originated from LAC populations^19^. Similarly, Abdill et al. surveyed over 168,000 amplicon-based gut profiles but identified only 1215 from LAC individuals—derived from just 11 studies^5^. Valderrama et al. more recently described the South American MicroBiome Archive (saMBA), which compiles over 3382 samples from 33 studies across South America^20^. Our own comprehensive literature review identified over 13,000 LAC-derived samples across more than 100 studies (Supplementary Table 1), revealing a larger body of work that is still fragmented and underutilized.

This emerging body of research has expanded the landscape of microbiome science. Studies in the region have helped define both the upper bounds of human microbial diversity and the rate at which it erodes under rapid lifestyle transitions^14,21^. Landmark studies of uncontacted and semi-nomadic communities—such as the Yanomami, Guahibo, and Matses—have revealed gut microbiota with extraordinary taxonomic richness and functional versatility^4,7,8^. These ancestral microbial consortia harbor gene pools involved in complex carbohydrate metabolism and xenobiotic resistance, and are largely absent in industrialized populations^21^. In contrast, urban cohorts in Buenos Aires, Bogotá, Lima, and São Paulo show a Prevotella-to-Bacteroides shift typical of processed Western diets, illustrating how globalized food systems homogenize microbiomes within a single generation^10–12,21,22^. Together, these findings position LAC as a unique setting for studying microbial “loss-of-function” trajectories and developing conservation-oriented microbiome frameworks.

A second major contribution is the region’s potential for innovative use of social and geographic contrasts to test ecological hypotheses that are difficult to address elsewhere. For instance, village-wide shotgun metagenomics in rural Honduras demonstrated that kinship, friendship, and co-residence explain strain-level microbial sharing more effectively than geography alone^17^. Similarly, longitudinal studies along Amazonian settlement gradients show that incremental market access, rather than simplistic urban–rural dichotomies, correlates with measurable declines in fiber-degrading microbial guilds and short-chain fatty acid (SCFA) production^23^. These finely resolved study designs move the field beyond coarse cross-sectional snapshots toward causal models that incorporate culture, economics, and social behavior.

LAC cohorts have also broadened the disease landscape in microbiome research. While studies on obesity and metabolic disease remain central^13,24^, LAC-based investigations uniquely examine endemic and neglected conditions such as parasitic coinfections^9,25^, post-infectious diarrhea^9,25^, and pediatric anemia^26,27^. These conditions can result in microbiome shifts more pronounced than those caused by BMI, ancestry, or diet alone. For example, in Ecuadorian children, pathogenic *Escherichia coli* infections result in long-term disruption of butyrate-producing taxa^28^. In Colombian adults, obesity explains more microbiome variation than genetic ancestry^13,29^. Such findings reinforce the need for locally derived baselines and context-specific reference models when designing microbiome-based diagnostics and therapies.

## Challenges and opportunities

### Structural gaps in capacity and infrastructure

**Fig. 1:**
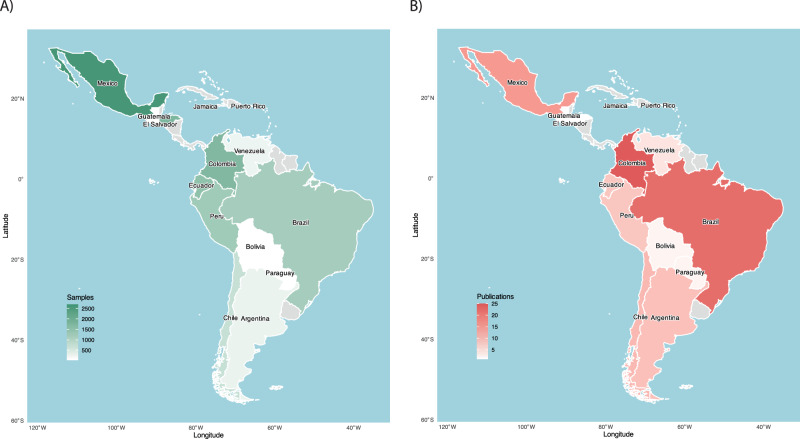
Geographic distribution of human gut microbiome studies in Latin America and the Caribbean (LAC). Number of samples (**A**) and publications (**B**) in human gut microbiome studies across Latin America and the Caribbean (LAC). It shows the asymmetry in the geographic distribution of microbiome research within LAC, with few countries dominating both sample collection and publications, mainly due to disparities in infrastructure, funding, and trained personnel. As a result, several countries have minimal or no representation, revealing a significant gap in regional equity in microbiome research. Generated from data in Supplementary Table 1. Maps were created using the rnaturalearth package in R (R version 4.5.1).

Despite growing interest in microbiome science, Latin America and the Caribbean remain underrepresented in global gut microbiome research due to persistent structural barriers. The region faces multiple challenges in infrastructure, funding, and trained personnel, which vary widely across countries and institutions. These disparities result in uneven sampling, publication output, and data quality across institutions and countries (Fig. 1). Most LAC-based studies rely on short-term, cross-sectional designs that are often underpowered and inadequately controlled for confounding variables. A shift toward well-powered, longitudinal designs with rigorous methodological frameworks is critical to increase both local and global scientific impact.

Cultural, dietary, and social diversity across the countries further demands context-specific methodologies. Standardized protocols and questionnaires imported from high-income settings often fail to capture relevant local variables. Instead, regionally tailored instruments (questionnaires, metadata structures, and sampling strategies) are needed to ensure meaningful data collection and culturally appropriate interpretations.

**Fig. 2:**
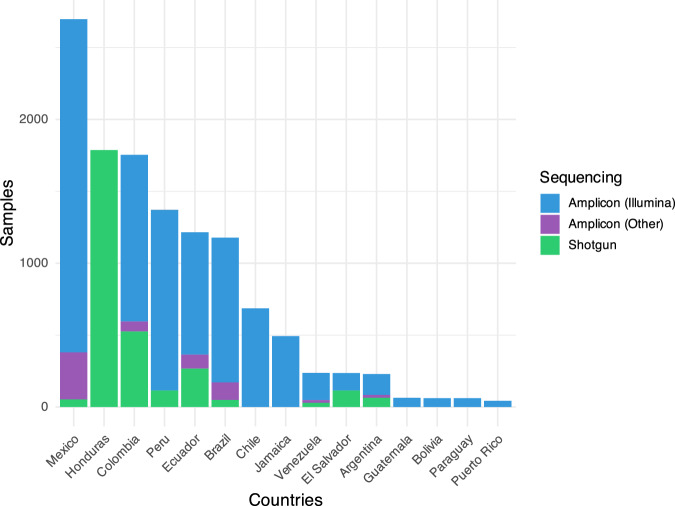
Human gut metagenomes by LAC country, divided by sequencing technology used. Amplicon sequencing, particularly via Illumina, is the predominant method used across LAC human gut microbiome studies, with shotgun metagenomics largely underrepresented. This suggests both cost and infrastructure barriers that limit comprehensive, high-resolution microbiome profiling in the region. Generated from data in Supplementary Table 1.

Resource constraints have also shaped the region’s technological output. Many LAC studies have relied on low-cost but outdated techniques such as amplicon sequencing with non-standard primers (Fig. 2). These methods limit comparability and taxonomic resolution with more recent studies, in particular from high-income countries, that increasingly adopt shotgun metagenomics, enabling functional profiling and strain-level resolution. Transitioning toward these global standards, while balancing cost-efficiency, is necessary to ensure data from LAC is interoperable and analytically robust.

### Building inclusive scientific capacity

A major barrier to equitable microbiome research in Latin America and the Caribbean (LAC) is the limited local expertise in genomics, bioinformatics, and microbial ecology. Many researchers still depend on foreign collaborators for sample processing, sequencing, data analysis, and data submission to public repositories, which weakens regional scientific leadership and undermines data sovereignty. Among 104 studies using LAC-derived samples, only 66% included a senior author affiliated with the region, and fewer than half deposited data in public repositories through LAC institutions (Supplementary Table 1). Inadequate training in metadata collection and sample preservation further leads to data loss or low-quality submissions, hindering the reusability of the data.

This capacity gap should be viewed as an opportunity. Regional training programs, South–South collaborations, and international exchanges can rapidly expand technical expertise. Strategic but modest investments in regional sequencing and bioinformatics centers would strengthen autonomy and shorten timelines for data processing and analysis. Nevertheless, high reagent costs and limited economies of scale will continue to constrain local sequencing capacity.

Inclusivity is also essential. Training and mentorship efforts should actively support women, Indigenous students, Afro-descendant communities, and other underrepresented groups to foster a microbiome research ecosystem that reflects the region’s social and cultural diversity. Building the capacity to frame questions, design experiments, and interpret findings from within the region is foundational for equity and long-term sustainability.

### Persisting funding challenges

LAC’s funding challenges are embedded in broader global inequities. In 2024, per capita GDP in LAC countries ranged from $10,000 to $17,000 USD, compared to $55,000 to $85,000 in North America and Europe^30^. Research and development (R&D) investments reflect this gap, with most LAC countries allocating less than 1% of GDP to science, according to the most recent data available for 2020, well below OECD standards^31^.

Many LAC nations are positioned in the upper limit of the LMIC (Low and Middle Income Countries) category, which presents a unique funding challenge: they are often overlooked by global foundations that prioritize lower-income regions, yet remain at a disadvantage when competing for high-level international grants. Moreover, national funding programs tend to focus on applied research tied to food, environment, or infectious diseases, leaving basic microbiome research severely underfunded. Addressing these funding challenges will require regional advocacy, targeted funding calls, and international investment strategies that recognize the immediate relevance of microbiome research for local public health, nutrition, and food security.

### Ethics and community partnership

Historical patterns of extractive research, particularly among Indigenous and rural communities, have led to justified skepticism regarding data misuse, unequal benefits, and exclusion from meaningful participation in science^32–34^. Addressing this requires co-designed research from the outset: projects must align with community-defined priorities and ensure relevance, reciprocity, and accountability.

Ethical frameworks in microbiome research should extend beyond compliance to ensure meaningful engagement with local communities. Researchers must plan and monitor tangible benefits, such as access to health interventions and training opportunities. Involving communities as partners by incorporating their priorities and traditional knowledge into research agendas is crucial. These frameworks should be collaboratively developed and include transparent benefit-sharing mechanisms aligned with international agreements like the Nagoya Protocol. Although well-intentioned, such frameworks can be challenging to implement and may inadvertently restrict research when misinterpreted. Sharing country-specific experiences can help create balanced policies that protect community rights while promoting scientific advancement.

Governance of biobanks and data repositories is especially critical. Oversight bodies that include scientists, community representatives, and ethics experts can ensure transparent decision-making and equitable access. Without such structures, even well-meaning research risks reproducing extractive dynamics.

### Data sovereignty and open science

Data sovereignty is critical for building lasting scientific leadership in the region. Microbiome data, once generated, can be shared and re-analyzed at low cost, making bioinformatics a robust and accessible tool for scientific discovery, even in settings with limited laboratory infrastructure. With appropriate training, open-source tools, and access to global datasets, local researchers can lead high-impact studies even with limited laboratory infrastructure.

However, datasets from the region are frequently deposited by foreign collaborators without proper metadata or local leadership. This undermines both reusability and equity. Ensuring that LAC scientists retain stewardship over locally generated data, including through shared authorship and leadership on secondary analyses, is vital.

Strengthening metadata standards, building robust regional data repositories, and adopting FAIR principles^35^ (Findable, Accessible, Interoperable, Reusable) will be critical to advancing LAC’s role in global microbiome science. High-quality metadata and consistent data curation will enhance the long-term value of datasets for reuse and cross-study comparisons. With appropriate infrastructure and supportive policies, the region is well-positioned to develop context-specific bioinformatic pipelines—for example, tools tailored to account for dietary diversity or parasitic infections, both highly relevant in tropical contexts. Strategic investment in data sovereignty and open science can enable LAC to establish a self-sustaining research ecosystem that supports both scientific innovation and equitable participation.

### Preserving microbial diversity

LAC harbors microbial diversity that is rapidly vanishing. Rapid modernization, globalization, and dietary Westernization are homogenizing lifestyles across Latin America and the Caribbean, placing the region’s microbial diversity at risk, particularly in rural, traditional, and Indigenous communities. As processed foods, antibiotics, and urban infrastructure reach increasingly remote areas, traditional microbiomes are shifting toward Westernized profiles, raising the possibility of irreversible loss of unique microbial taxa within a single generation. Preserving this microbial heritage is critical, not only for its cultural and ecological value, but also for its potential to inform future therapeutic discoveries. Concerted efforts are needed to document, sample, and biobank these microbiomes, exemplified by initiatives such as the Microbiota Vault^36^ and the Global Microbiome Conservancy^37^. Just as linguistic and ecological diversity warrant protection, so too does microbial diversity before it is altered or lost. Equally important, communities must retain ownership and control over their biological resources and their downstream applications, ensuring that preservation efforts are grounded in ethical and equitable scientific practices.

## The Latinbiota Consortium

To address the structural challenges facing microbiome research in Latin America and the Caribbean and to harness the region’s scientific potential, a regionally led, collaborative network dedicated to advancing equitable microbiome research began in 2019 with 22 researchers from eight LAC countries, working in partnership with the Wellcome Sanger Institute (UK) to explore gut microbiome diversity across regional populations^38^. Building up from that collaborative effort, we now introduce the *Latinbiota Consortium*.

The consortium’s first phase involved deep sequencing of over 600 samples collected from participating countries (Argentina, Bolivia, Brazil, Colombia, Chile, Ecuador, Mexico and Uruguay), a process that highlighted the promise and complexity of regional microbiome research. Significant logistical and administrative barriers had to be addressed, including the harmonization of sampling and metadata protocols, navigation of local permitting systems, and compliance with the Nagoya Protocol. Limited institutional and administrative support further slowed progress. Despite these obstacles, the project succeeded in generating one of the largest LAC-specific microbiome datasets to date. The analysis of this dataset is nearing completion and has yielded valuable operational and ethical lessons for other consortia working in under-resourced or diverse geopolitical contexts.

Looking ahead, Latinbiota aims to develop into a sustainable platform that coordinates microbiome research across LAC, connecting efforts from grassroots sampling to cross-national, multi-institutional studies. The overarching goal is to characterize, preserve, and study the region’s microbial diversity through an inclusive, ethical, and high-impact research agenda led by LAC scientists and communities. Key priorities include:

- **Fostering regional connectivity and collaboration**: Establish a formal network of microbiome researchers across countries and disciplines to facilitate communication, collaborative grant writing, shared infrastructure, and pooled expertise.
- **Standardizing context-appropriate methodologies**: Develop and disseminate harmonized protocols for sample collection, sequencing, and metadata reporting, tailored to local contexts. This will enable cross-study comparisons, meta-analyses, and data integration at regional and global scales.
- **Embedding ethical co-design from the outset**: Work with Indigenous representatives, ethics committees, and social scientists to create culturally responsive research frameworks that address informed consent, community priorities, result sharing, and equitable benefit distribution.
- **Strengthening biobanking and sequencing capacity:** Coordinate the use of regional infrastructure for sample storage and sequencing, with attention to cost-effectiveness, data sovereignty, and sustainability.
- **Promoting data stewardship and shared analysis:** Build or leverage regional platforms for data storage, curation, and access, ensuring that LAC researchers retain visibility, authorship, and analytical leadership. Co-analysis will be fostered through training workshops, collaborative publications, and bioinformatics hackathons.
- **Enabling equitable international collaboration:** Serve as a structured gateway for global partners to engage with LAC-led microbiome research through respectful and mutually beneficial collaborations. Latinbiota will ensure that international partnerships contribute to local capacity-building while reinforcing regional leadership.

Through these actions, Latinbiota aims to address historical inequities in microbiome science while establishing Latin America and the Caribbean as a global leader in research on microbial diversity. This initiative is guided by regional priorities, led by local researchers, and grounded in ethical engagement with the communities involved.

## Reclaiming microbial futures

As microbiome science expands globally, it must do so inclusively, ensuring that no region is left behind. Latin America and the Caribbean represent not only a critical gap in global microbiome datasets but also a region of exceptional scientific creativity. Researchers across LAC are already advancing the field through high-impact studies that map ancestral microbial diversity, document the rapid effects of urbanization, investigate socially structured microbial transmission, and explore the microbiome’s role in endemic disease dynamics. These efforts illustrate how locally grounded science can address globally relevant questions.

As highlighted throughout this Perspective, the region’s ethnogeographic diversity, complex epidemiological transitions, and rich traditional knowledge position LAC as indispensable to microbiome research. Supporting this work is not only a matter of equity; it is essential for discovery. Large-scale, collaborative initiatives rooted in the region could uncover novel microbial functions and interactions that remain invisible in datasets from high-income settings. Integrating microbiome science with local health priorities and ancestral knowledge holds the potential to generate culturally grounded, context-specific interventions for diverse urban and rural populations.

The urgency is clear: the microbiomes of LAC are changing rapidly. Without action, the erosion of microbial diversity caused by urbanization, globalization, and dietary shifts may soon become irreversible. Now is the moment to invest in this field; not just financially, but intellectually, ethically, and collaboratively. The global research community must engage with LAC not merely as a source of samples but as an equal partner and leader in shaping the future of microbiome science.

We envision a future in which a young scientist in Kingston or Bogotá launches a microbiome study with the same visibility and support as their peers in Boston or London; where children in rural Haiti or the Peruvian Andes benefit from microbiome-informed health interventions tailored to their communities; and where transformative discoveries emerge just as readily from Buenos Aires or San José as from Silicon Valley. Realizing this vision demands more than funding; it requires solidarity, sustained collaboration, and a fundamental reimagining of how science is practiced, led, and shared.

The Latinbiota Consortium invites researchers, institutions, and stakeholders around the world to join us in this collective effort. Together, we can preserve and harness the microbial heritage of Latin America and the Caribbean to improve lives across the region and to advance scientific knowledge that will resonate globally for generations to come. Researchers interested in participating are encouraged to contact the corresponding authors.

## Supplementary information

Supplementary Table 1
